# Cognate peptide-receptor ligand mapping by directed phage display

**DOI:** 10.1186/1477-5956-3-7

**Published:** 2005-06-17

**Authors:** Thomas Stratmann, Angray S Kang

**Affiliations:** 1 Department of Molecular Biology, The Scripps Research Institute, 10550 North Torrey Pines Road, La Jolla, CA 92037, USA; 2 Universidad de Barcelona, Departamento de Fisiologia, Diagonal 645, 3°, 08028 Barcelona, Spain

## Abstract

**Background:**

A rapid phage display method for the elucidation of cognate peptide specific ligand for receptors is described. The approach may be readily integrated into the interface of genomic and proteomic studies to identify biologically relevant ligands.

**Methods:**

A gene fragment library from influenza coat protein haemagglutinin (HA) gene was constructed by treating HA cDNA with DNAse I to create 50 – 100 bp fragments. These fragments were cloned into plasmid pORFES IV and in-frame inserts were selected. These in-frame fragment inserts were subsequently cloned into a filamentous phage display vector JC-M13-88 for surface display as fusions to a synthetic copy of gene *VIII*. Two well characterized antibodies, mAb 12CA5 and pAb 07431, directed against distinct known regions of HA were used to pan the library.

**Results:**

Two linear epitopes, HA peptide 112 – 126 and 162–173, recognized by mAb 12CA5 and pAb 07431, respectively, were identified as the cognate epitopes.

**Conclusion:**

This approach is a useful alternative to conventional methods such as screening of overlapping synthetic peptide libraries or gene fragment expression libraries when searching for precise peptide protein interactions, and may be applied to functional proteomics.

## Background

Conventional approaches to elucidate peptide protein interactions include the screening of synthetic sequentially overlapping peptides [1,2], peptide fragments created by enzymatic digestion [3] or expressed gene fragments created by recombinant DNA technologies [4]. For more than a decade, phage display technology has been applied to elucidate protein-protein interactions. Random peptide libraries have been useful to predict epitope sequence mimics of unknown ligands. The epitopes for antibodies have been predicted from consensus sequences derived from aligning motifs with the original protein sequence of interest [5,6]. When the original ligand is not known, it is possible to predict potential binding consensus sequences. Castaño and colleagues predicted a binding motif for murine CD1 using a 22 amino acid random library [7]. In some instances, the ligand and receptor are known and the precise interactions between the two can be determined empirically as outlined above. A semi-empirical approach involves displaying randomly generated fragments of the target genes on fd phage [8]. The method has been used to elucidate epitopes for mAbs directed against the bluetongue virus outer capsid protein [9], human plasminogen-activator inhibitor 1 [10], large subunit of *Drosophila *RNA polymerase II, human p53, and the human cytokeratin 19 [11].

In this study, we report the epitope mapping of a monoclonal and polyclonal antibody directed against the influenza coat protein HA by a two-step approach. First, the target cDNA is digested randomly with DNAse I and cloned into a pre-selection plasmid Open Reading Frame Expression and Secretion (pORFES IV), described in US Patent 6,586,236 [12], between the ompA leader sequence and the β-lactamase gene. The leader sequence directs peptide-β-lactamase fusion to the periplasm of *E. coli*.

Fragments, which do not restore the open reading frame of the β-lactamase, as well as non-secreting inserts, are eliminated by selectively propagating in the presence of carbenicillin. Secondly, the selected inserts are transferred to a phage surface display vector JC-M13-88 [13,14] for display on p8 (encoded by a synthetic copy of gene *VIII*). The method allows the construction of libraries from complex systems (mix of cDNAs) or possibly genomic DNA. The approach demonstrated here allows rapid and precise mapping of epitopes recognized by antibodies using cDNA encoding the target protein to construct an epitope display library.

## Results

### Construction of a random haemagglutinin fragment epitope library as a β-lactamase fusion protein

**Figure 1:**
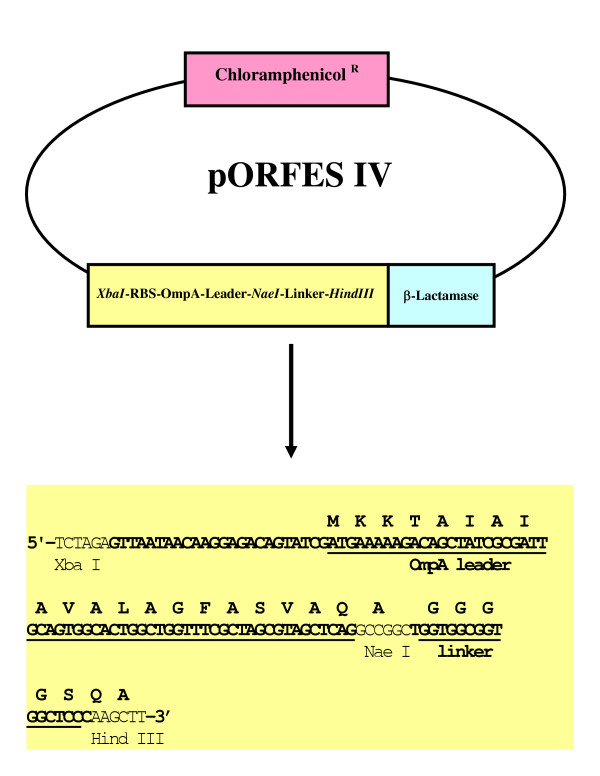
**Insert modification of pORFES to pORFES IV to permit cloningof blunt-ended fragments**. The original pORFES [12, 14] vector was designed to accept inserts as *NheI-HindIII *fragments. To create pORFES IV, the *NheI *– *HindIII *stuffer was replaced with aninsert to include a *NaeI *site, an additional bp and a (Gly)_4 _spacer. The ligation of inserts restoring the correct frame of the β-lactamase confers carbenicillin resistance.

An epitope fragment library from the influenza HA gene (influenza X-31 [H3N2], [15]) was generated by randomly digesting the HA DNA with DNAse I. The conditions were adjusted in order to create predominantly 50 – 100 bp fragments that were subsequently cloned into the modified pORFES vector [12]. The "empty" pORFES IV vector contains an ompA leader followed by the β-lactamase gene, which is out of frame as shown in Figure 1. Insertion of DNA fragments at the *NaeI *site that restore the reading frame between the ompA leader and the β-lactamase coding sequence, permits secretion and allows for positive selection using β-lactam antibiotics. *E. coli *XLOLR electrocompetent cells were transfected with the ligation products. After 1-hour recovery at 37°C, aliquots of the cells were plated on LB agar plates containing either chloramphenicol alone or in combination with carbenicillin. Since cell growth in the presence of carbenicillin requires restoration of the β-lactamase reading frame, colonies growing on chloramphenicol alone served as a representation of the total number of transformation events regardless of the presence or nature of the insert (i.e., containing either the self-ligated vector without an insert, or the vector ligated with an insert but not necessarily in-frame with β-lactamase). As expected, approximately 1/3 of the transformation events resulted in restoring the correct reading frame. The size of the primary HA library in pORFES was determined by plating on agar plates containing carbenicillin and chloramphenicol.

Approximately 1.7 × 10^6 ^independent clones were generated. Since the insert could have one of two possible orientations in three reading frames, and since some of these fragments may have included stop codons, theoretically less than 1/6 of all clones should encode cognate HA peptides. Based on this assumption, the library size for cognate HA peptides was estimated to contain approximately 2.8 × 10^5 ^independent clones.

### Cloning of the haemagglutinin fragment library into JC-M13-88

Amplification of the HA fragment library in pORFES IV permitted the positive selection of open reading frames and provided ample material for directional cloning into the display vector JC-M13-88. The unique restriction sites, *XbaI-HindIII*, facilitated directional transfer of the library from pORFES IV into JC-M13-88. These *XbaI-HindIII *fragments contain the ribosome-binding site, the ompA leader sequence followed by the random insert of the library. Although the DNA digestion yielded mainly 50 – 100 bp fragments, larger fragments were also found in the pORFES vector. In order to obtain only peptides of a length of about 15 to 30 amino acids, the *XbaI-HindIII *fragments were resolved on a TBE gel and DNA fragments with the appropriate size eluted. Cloning the libraries into JC-M13-88 afforded 6 × 10^6 ^independent clones for the HA phage library. This figure is ten-fold higher than the original pORFES IV HA library size. This over-representation ensures that most of the library members are transferred to the display system.

### Panning of the haemagglutinin fragment library against mAb 12CA5

**Figure 2:**
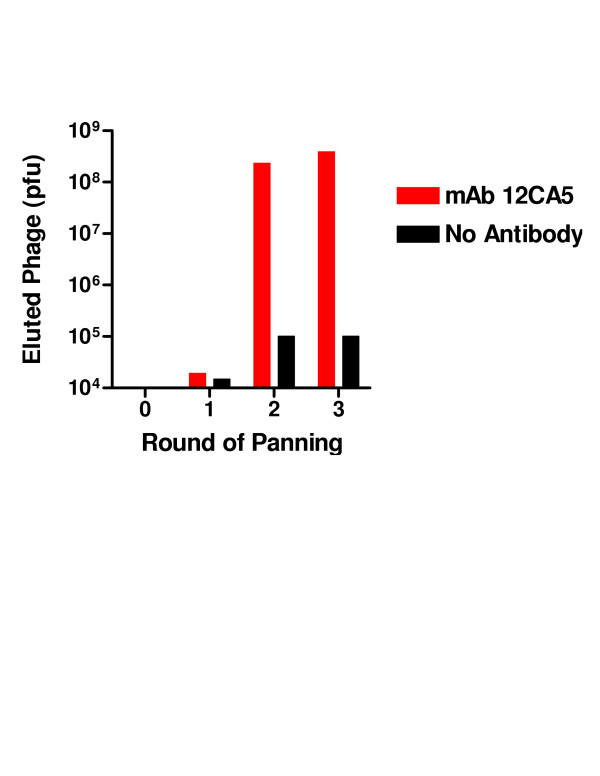
**Panning of the HA fragment phage library with mAb 12CA5**. The HA fragment phage display library was selected against the anti-haemagglutinin mAb 12CA5 or wells coated with BSA (no antibody). The eluted phage was quantified after each round of panning by plaque assay.

**Figure 3:**
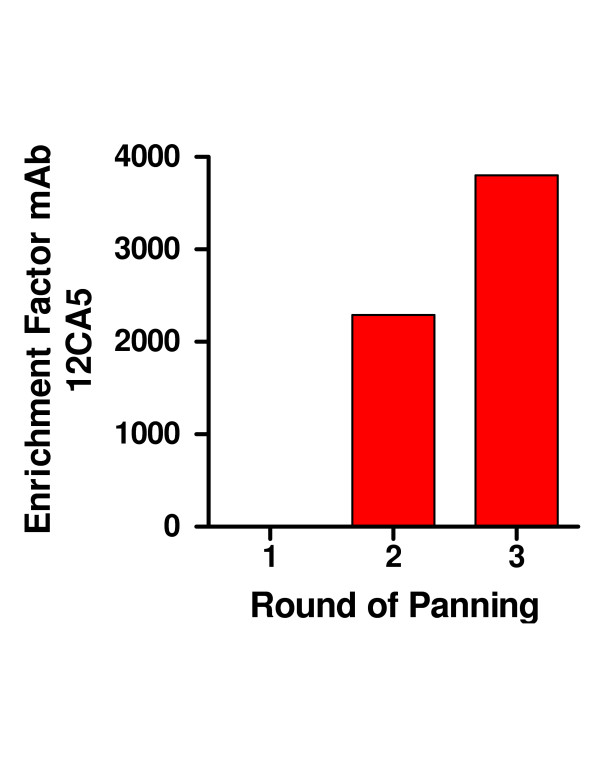
**Panning of the HA fragment phage library with mAb 12CA5**. The enrichment factor was calculated as the ratio of eluted phage from wells coatedwith antibody or BSA only, respectively, for each round of panning

**Figure 4:**
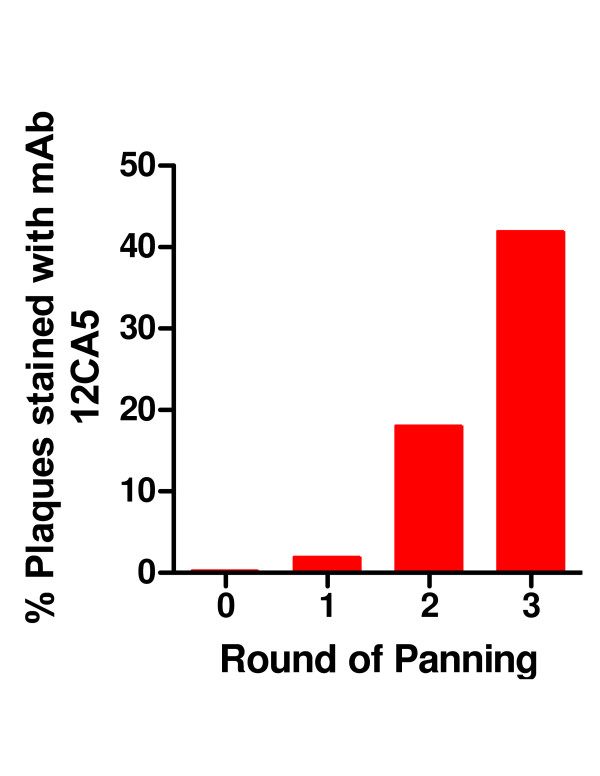
**Panning of the HA fragment phage library with mAb 12CA5**. An aliquots of eluted phage after each round of panning were analyzed by filter lifts for binding to mAb 12CA5. The filters were blocked with BSA, incubated with mAb 12CA5 and subsequently detected with goat anti-mouse kappa Ab coupled to alkaline phosphatase.

The well-characterized mAb 12CA5 that binds to the HA sequence YPYDVPDYAS [16] was used to screen the initial HA library prior to panning in order to determine the initial frequency of the cognate peptide fragment. Approximately 4100 plaques of the naïve (i.e. prior to panning) library were analyzed by probing filter lifts with mAb 12CA5. Initially, only 0.4% plaques were immunoreactive. This number increased with each round of selection. The library was subjected to 3 rounds of panning against mAb 12CA5. The second round of panning resulted in a 12,000-fold increase of eluted phage (2300 fold over background). Approximately 18% of the plaques stained strongly with mAb 12CA5. This number increased to 42% after the third round of selection (Figures 2, 3, 4). Phage from 6 stained plaques were isolated and the insert DNA sequence determined. The results are shown in Table 1 (see additional file 1). Half the clones were identical whereas the other 3 clones differed from each other. All 6 clones contained the YPYDVPDYAS motif.

### Panning of the haemagglutinin fragment library against pAb 07431

**Figure 5:**
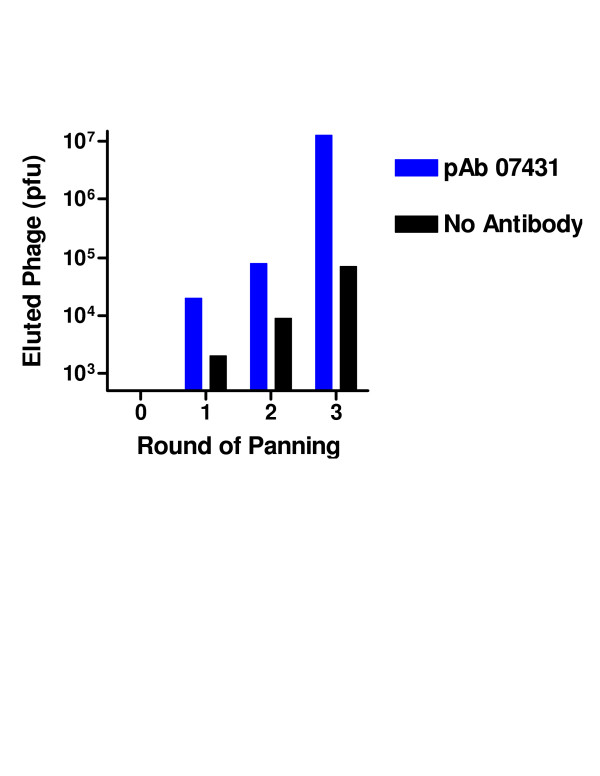
**Panning of the HA fragment phage library with pAb 07431**. The HA fragment phage display library was selected against the anti-haemagglutinin pAb 07431 or wells coated with BSA only (no antibody). The Eluted phage was quantified after each round of panning by plaque assay.

**Figure 6:**
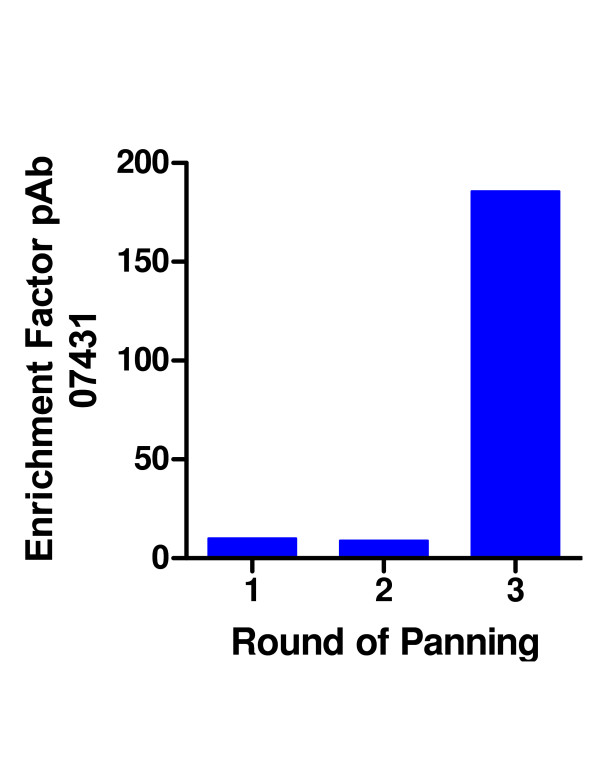
**Panning of the HA fragment phage library with pAb 07431**. The enrichment factor was calculated as the ratio of eluted phage from wells coated with pAb 07431 or BSA only, respectively.

**Figure 7:**
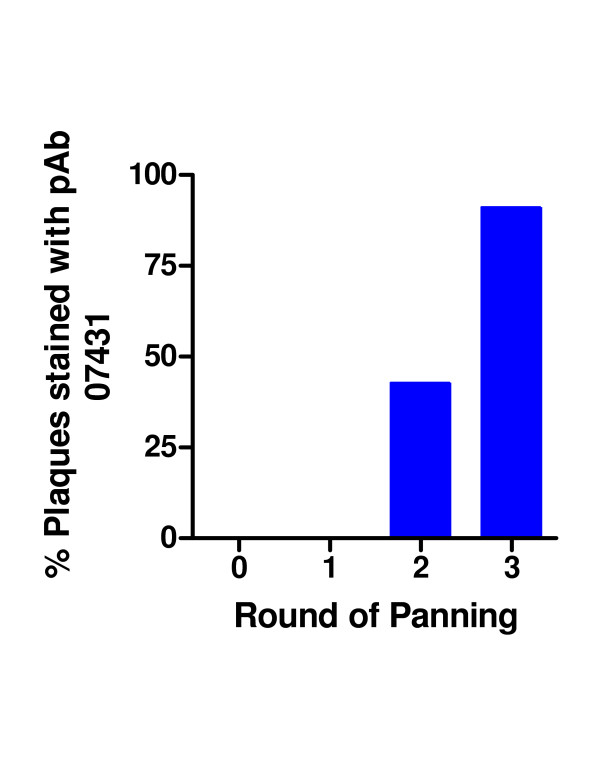
**Panning of the HA fragment phage library with pAb 07431**. An aliquots of eluted phage after each round of panning were analyzed by filter lifts for binding to pAb 07431. The filters were blocked with BSA, incubated with pAb 07431 and subsequently detected with goat anti-rabbit IgG Ab coupled to alkaline phosphatase.

Subsequently, the library was evaluated using a rabbit polyclonal IgG raised against the peptide CKRGPDSGFFSRLNWLYKSG. When 2400 plaques of the initial library were screened by filter lift for immunoreactive clones, a higher background staining was observed relative to filter lifts stained with mAb 12CA5. However, one plaque was found to stain strongly with the pAb 07431. During selection, the number of eluted phage increased with each round. After the third round, 91% of plaques bound to pAb 07431 as determined by filter lifts (Figures 5, 6, 7). A sequence analysis of 16 clones revealed only 3 different sequences as shown in Table 2 (see additional file 2). All of the selected sequences contained the GFFSRLNWLTKS motif that is part of the peptide used for the immunizations. The immunizing peptide was derived from influenza strain X47 (HA1, [17]) and differed by a single substitution of T to Y from the gene used for this study (derived from influenza X-31 [H3N2]) as shown in Table 2. Despite this point variation the approach permitted recovery of the epitope.

Interestingly, though, clones TSS 399 and 401–403 encoded additional sequences resulting from frame shifts and a multiple insertion event. A BLAST search of the nucleotide sequence encoding the TSS 399 peptide on phage (5' – **TCA TGT GGG CCT GCC AGA GAG GCA ACA TTA GGT GCA ACA TTT GCA TTT GAG TGT **GGT AGC GGT TTT TTC AGT AGA CTG AAC TGG TTG ACC AAA TCA-3') in GenBank (access code V01103), revealed that nucleotide1–54 (shown in bold) aligned with nucleotides 1681–1734 of HA (incorrect frame). As expected, a search with the corresponding encoded 18-mer peptide (SCGPAREATLG ATFAFEC) failed to align on the HA polypeptide. Nucleotides 57–96 encoding the cognate epitope aligned with HA nucleotides 509–548 (in frame).

## Discussion

Here we describe an approach for the mapping of linear peptide epitopes of fragmented genes by phage display. The procedure differs notably from other approaches of phage display of targeted gene fragments, as functional inserts are preselected prior to phage display. The vector pORFES IV (plasmid Open Reading Frame Expression and Secretion, [12]) permits the positive selection of fragments that give rise to open reading frames and allow expression of β-lactamase. The vector is designed such that ompA leader sequence cleavage occurs immediately prior to the insert. The advantage of such positioning of the cloning and cleavage site is that the insert encodes the N-terminus of the displayed polypeptide, not the vector. This may be important if the ligand requires a free N-terminus for receptor binding. By selecting bacteria in the presence of carbenicillin we are able to rescue plasmids with functional inserts and transfer fragments of desired size into the phage display vector. Conventional phage display libraries contain a mixture of functional and nonfunctional inserts along with phage without inserts. The propagation of phage without inserts or with non-functional inserts is favored, which affects phage library composition, reducing the functional complexity of the library. This qualitative step ensures a greater population of the initial phage libraries encode and display peptide. Approximately 87% of all primary transformation events were eliminated using this pre-selection step, implying that only 13% of the transformation events were functional. This figure is in accordance with the predicted number of potential open reading frames generated by this approach. This also highlights the degree of redundancy in screening conventional libraries for expressed sequences. The preselection step does not preclude the occurrence and accumulation of deletions or frame shifts during phage propagation. However, since the initial starting library is highly functional (i.e., it contains only phage displaying peptides), these acquired deletions or frame shifts should not significantly impact on the subsequent use of the library.

We validated the semi-empirical approach to targeted phage display by constructing a random epitope library from the gene encoding influenza virus protein HA. The DNA was digested and purified by agarose gel electrophoresis to create fragments encoding peptides of 15 to 35 amino acids. Affinity selection was carried out using a mAb and a pAb, which had been created by immunizing a mouse and a rabbit, respectively, with synthetic peptides representing short HA sequences. Thus, at the on-set of these experiments, the epitopes recognized by these antibodies were known. By subjecting the library to three rounds of selection against either of the antibodies, we were able to retrieve both sequences from the library. Panning against mAb 12CA5 revealed a 13 amino acid long consensus sequence, only 3 amino acids longer than the reported epitope for this Ab. When the library was panned against pAb 07431, a consensus sequence of 12 amino acids of length was found, representing the HA peptide 162–173 which is contained by the peptide used for immunization (HA 157–178).

Since we retrieved two distinct epitopes from this random epitope library, it is reasonable to assume that a large representation of open reading frames had been generated and displayed. In addition to selecting the pAb 07431 epitope, clone TSS 399 also encoded additional sequences that also produced an in-frame peptide. Simple BLAST analysis of the nucleotide and peptide sequences allowed us to determine the source of the additional sequence. The BLAST searches revealed that two inserts in tandem had been cloned into the vector, the first out of frame and the second insert inframe, resulting in a chimeric peptide containing the cognate sequence.

Random fragment epitope phage display libraries in combination with affinity selection are very useful tools to find cognate ligands to monoclonal or polyclonal antibodies, at least for linear epitopes. The method described here utilizes a preselection step to enrich for sequences with open reading frames that are readily secreted as fusion proteins with β-lactamase, a prerequisite for subsequent phage display. This ensures that a greater proportion of the phage library not only encodes a polypeptide, but that it also has the potential to display it. This approach to building a phage display peptide library has two key features; first N-terminus of the displayed peptide is encoded by the insert, and secondly that the displayed peptides are approximately 15–35 amino acids in length. This is critical if selecting for peptides binding to MHC class I or II molecules. The approach has been successfully applied for the selection of peptide epitopes derived from ovalbumin and glutamic acid decarboxylase specific for MHC class II molecules [18,19]. This technology might very well be successfully extended to more complex systems in which several cDNAs encoding up-regulated tumor associated genes are processed for epitope screening against antibodies, MHC class I or II molecules [18], or in other protein-protein interactions.

## Conclusion

With the speed of library generation from either genomic and cDNA outpacing the emerging field of proteomics, a backlog in correlating gene to function and mapping protein-protein interactions is creating a bottleneck. Here, we hint at a possible route to assist in elucidating the interacting biologically relevant motifs directly from DNA. The example described utilized two antibodies (proteins) and an antigen encoding nucleic acid (DNA) to develop a proof of concept. Additionally, this approach has also been applied to elucidation of GAD peptides binding to MHC class II I-Ag^7 ^molecules [18]. Once a key protein has been identified, it may be possible to use genetic information and selection to identify cognate ligands.

The use of pORFES IV to eliminate "junk" sequences enhances the quality of the displayed library, resulting in enhanced panning efficiency. This approach has already been applied to improving antibody fragment libraries [14], and may also be applied to creating higher quality random peptide display libraries.

## Methods

### Monoclonal and polyclonal antibodies

The mAb 12CA5 specific for a HA peptide (YPYDVPDYAS)[16] and the rabbit polyclonal serum 07431 against the peptide (CKRGPDSGFFSRLNWLYKSG) derived from Influenza X-47 HA1 were kindly provided by Dr. R. A. Lerner (The Scripps Research Institute). The rabbit IgG fraction was purified from the sera using protein A-sepharose (Pierce, Rochford, IL, USA).

### Plasmids and bacterial strains

The plasmid Open Reading Frame Expression and Secretion IV is based on(pORFES) [12,14] with an insert modification shown in Figure 1. The phage display vector JC-M13-88 has been described elsewhere [12-14]. The host strains *Escherichia coli *XLOLR and XL1-Blue were purchased from Stratagene (La Jolla, CA, USA). Plasmid pCMU encoding HA cDNA (derived from influenza X-31 [H3N2], [15]) was a kind gift from Dr. G. Aichinger (Hammersmith Hospital, London, UK). All enzymes were purchased from Boehringer-Mannheim (Indianapolis, IN, USA).

### Haemagglutinin gene fragmentation process

The plasmid pCMU (100 μg) was digested with *SmaI/XbaI *to release the HA coding insert. The gel-purified inserts (~10 μg DNA) was digested with 70 ng of DNAse in 50 mM Tris-HCl, pH 7.2, containing 40 mM MnCl^2 ^(130 μl) for 4 minute sat 37°C and the reaction was terminated by adding EDTA to a final concentration of 70 mM. After precipitation, the fragments were treated with 0.001 units mung bean nuclease per μg DNA for 5 minutes at 37°C. The resulting mixtures of blunt ended fragments were ligated between the ompA leader sequence and the β-lactamase gene into pORFES IV that had been digested with *NaeI *and treated with calf intestine phosphatase. The ligation products were transfected into non-suppressing *E. coli *XLOLR via electroporation and propagated overnight in Super Broth containing 100 μg/ml carbenicillin at 37°C. The inserts that restore the reading frame and that are readily translocated into the periplasm along with the fused β-lactamase confer β-lactam antibiotic resistance. The transfection efficiency was monitored by plating aliquots on agar plates containing 25 μg/ml of chloramphenicol with or without 100 μg/ml carbenicillin. The pORFES IV library DNA was recovered from the overnight culture and inserts released by digestion with *XbaI *and *HindIII*. The fragments of 160–210 bp were resolved on a 3% agarose TBE gel.

### Preparation of M13 HA fragment display library

The 160–210 bp *XbaI/HindIII *fragments encode a ribosome binding site, the ompA leader and the HA derived inserts. These were directionally cloned into the phage vector JC-M13-88, between a *lac *promoter and a synthetic copy of gene VIII. The ligation products were transfected into XL1-Blue *E. coli *cells via electroporation and the transfection efficiency monitored by plaque assay. The phage were propagated for 16 h at 37°C in Super Broth containing 1 mM isopropyl-β-D-thiogalactopyranoside (IPTG) and isolated by PEG precipitation twice following standard methods [20]. The phage pellets were resuspended in phosphate buffered saline (PBS) and stored at 4°C.

### Panning

The JC-M13-88 library containing the HA gene fragments was panned against mAb 12CA5 and polyclonal rabbit IgG 07431. Multi-well maixisorp strips (Nalge Nunc International, Naperville, IL USA) were coated with 100 μl of a 8 μg/ml antibody solution in PBS or with 5 mg/ml BSA in PBS (negative control) over night at 4°C. The wells were washed 3 times with PBS containing 0.1% Tween (PBST), blocked with 0.5% BSA in PBS for 1 h at 37°C and washed as before. Approximately 10^10 ^plaque forming units (pfu) were added in 100 μl of PBS containing 0.25% BSA and 0.05% Tween. The panning was carried out in duplicate. After incubation for 90 minutes at 37°C, the wells were washed 10 times with PBST, the phage eluted with 100 μl of 100 mM glycine, pH 2.2, at ambient temperature for 10 minutes and the solution neutralized by adding 10 μl of 1 M Tris solution (pH not adjusted). An aliquot of the solution was retained to assess the phage output and the rest was used to infect *E. coli *XL1-Blue cells for propagation as described above.

### Filter lifts

To determine the relative abundance of individual binding clones, filter lifts were carried out on the library prior to panning, and subsequently after each round of panning. Phage plaques were overlaid with nitrocellulose filters for 10 minutes at 37°C. The filters were subsequently removed and blocked with 0.5% BSA in PBS for 1 h at RT and washed 3 times with PBST. The mAb 12CA5 and pAb 07431 were adjusted to 2 μg/ml PBST and the filters incubated with the Ab solution for 1 h at RT. The filters were washed as before and incubated with a goat anti-mouse antibody (Southern Biotechnology Associates, Inc., Birmingham, AL USA), or a goat anti-rabbit IgG F(ab)2 fragment (United States Biochemical Corp., Cleveland, OH, USA) conjugated to alkaline phosphatase. Both secondary antibodies were diluted in PBST(1 μg/ml final concentration) and the filters incubated for 1 h at RT. After washing as before, the filters were stained with 5-bromo-4-chloro-3-indolylphosphate (BCIP) and 4-nitro blue tetrazolium chloride (NBT).

### Analysis of individual clones

Individual phage plaques were picked and grown overnight after infection with *E. coli *XL1-Blue cells [20]. The double stranded replicative form (RF) DNA template was sequenced.

## Competing interests

The author(s) declare that they have no competing interests.

## Disclaimer

The views and opinions expressed herein are those of the authors and do not purport to reflect those of the Universidad de Barcelona.

## Authors' contributions

TS was the graduate researcher on this project. ASK was the PI.All authors read and approved the final manuscript.

## Supplementary Material

Additional File 1 **Table 1 – Alignment of haemagglutinin amino acids 112–133 with 6 peptide sequences displayed on JC-M13-88 after panning against mAb 12CA5. **Six clones of the HA phage display library were analyzed after three rounds of panning with mAb 12CA5. All clones showed a positive reaction in a filter lift using mAb 12CA5. Three clones were identical, and all the clones contained the consensus sequence YPYDVPDYAS against which the mAb is directed (in red bold letters). Click here for file

Additional File 2 **Table 2 – Alignment of haemagglutinin amino acids 157–178 with 15 peptide sequences displayed on JC-M13-88 after panning against the polyclonal rabbit IgG 07431. **15 clones of the HA phage display library were analyzed after three rounds of panning with pAb 07431 raised against the haemagglutinin derived peptide CKRGPDSGFFSRCNWLYKSG. All clones showed a positive reaction in a filter lift using pAb 07431. Several clones were identical and 3 different sequences were identified. All the clones contained the consensus sequence GFFSRLNWLTKS (in blue bold letters). Click here for file
