# Supplementary material for: Cognate peptide-receptor ligand mapping by directed phage display
Source: Proteome Sci. 2005 Jun 17;3:7. doi: 10.1186/1477-5956-3-7 (PMC1183247; doi:10.1186/1477-5956-3-7)
Supplement: Additional File 1 — Table 1 – Alignment of haemagglutinin amino acids 112–133 with 6 peptide sequences displayed on JC-M13-88 after panning against mAb 12CA5. Six clones of the HA phage display library were analyzed after three rounds of panning with mAb 12CA5. All clones showed a positive reaction in a filter lift using mAb 12CA5. Three clones were identical, and all the clones contained the consensus sequence YPYDVPDYAS against which the mAb is directed (in red bold letters). [file 1477-5956-3-7-S1.pdf]

Encoded Peptide	mAb 12CA5 Selected Phage
CYPYDVPDYASLRSLVASSGTL	**Cognate HA Peptide 112-133**
YPYDVPDYASLRSLVASSGTL	**TSS 397**
CYPYDVPDYASLRSLVASSG	**TSS 395**
CYPYDVPDYASLRS	**TSS 396**
CYPYDVPDYASLRSL	**TSS 391**
CYPYDVPDYASLRSL	**TSS 392**
CYPYDVPDYASLRSL	**TSS 394**
