# Supplementary material for: Cognate peptide-receptor ligand mapping by directed phage display
Source: Proteome Sci. 2005 Jun 17;3:7. doi: 10.1186/1477-5956-3-7 (PMC1183247; doi:10.1186/1477-5956-3-7)
Supplement: Additional File 2 — Table 2 – Alignment of haemagglutinin amino acids 157–178 with 15 peptide sequences displayed on JC-M13-88 after panning against the polyclonal rabbit IgG 07431. 15 clones of the HA phage display library were analyzed after three rounds of panning with pAb 07431 raised against the haemagglutinin derived peptide CKRGPDSGFFSRCNWLYKSG. All clones showed a positive reaction in a filter lift using pAb 07431. Several clones were identical and 3 different sequences were identified. All the clones contained the consensus sequence GFFSRLNWLTKS (in blue bold letters). [file 1477-5956-3-7-S2.pdf]

Encoded Peptide	pAb 07431 Selected Phage
**GPDSGFFSRLNWLYKSG**STYPV	**Cognate HA X47 157-178**
GPDS**GFFSRLNWLTKS**GSTYPV	**HA X31 157-178 cDNA**
SCGPAREATLGATFAFECGS**GFFSRLNWLTKS**	**TSS 399**
SCGPAREATLGATFAFECGS**GFFSRLNWLTKS**	**TSS 401**
SCGPAREATLGATFAFECGS**GFFSRLNWLTKS**	**TSS 402**
SCGPAREATLGATFAFECGS**GFFSRLNWLTKS**	**TSS 403**
**GFFSRLNWLTKS**GST	**TSS 400**
GPGS**GFFSRLNWLTKS**GSTYPVP	**TSS 404**
GPGS**GFFSRLNWLTKS**GSTYPVP	**TSS 405**
GPGS**GFFSRLNWLTKS**GSTYPVP	**TSS 406**
GPGS**GFFSRLNWLTKS**GSTYPVP	**TSS 407**
GPGS**GFFSRLNWLTKS**GSTYPVP	**TSS 408**
GPGS**GFFSRLNWLTKS**GSTYPVP	**TSS 409**
PGS**GFFSRLNWLTKS**GSTYPVP	**TSS 410**
GPGS**GFFSRLNWLTKS**GSTYPVP	**TSS 411**
GPGS**GFFSRLNWLTKS**GSTYPVP	**TSS 412**
GPGS**GFFSRLNWLTKS**GSTYPVP	**TSS 413**
